# Analysis of postoperative reintervention for thoracoabdominal aortic aneurysm treated with fenestrated/branched endovascular repair

**DOI:** 10.3389/fcvm.2025.1530974

**Published:** 2025-05-27

**Authors:** Zhaohui Pan, Dongsheng Fu, Jianhang Hu, Yuexue Han, Zhao Liu

**Affiliations:** Department of Vascular Surgery, Nanjing Drum Tower Hospital, Affiliated Hospital of Medical School, Nanjing University, Nanjing, China

**Keywords:** fenestrated or branched endovascular aortic repair (F/B EVAR), aneurysm, reintervention, complication, endoleak

## Abstract

**Objective:**

Fenestrated/branched endovascular repair (F/B EVAR) has emerged as a frontline treatment for complex aortic diseases, yet multiple studies have reported high reintervention rates postoperatively. This study aims to discuss strategies for improving patient prognosis by analyzing the reintervention status following F/B EVAR in a single-center patient cohort.

**Methods:**

This is a single-center retrospective study that collected clinical data and follow-up information from patients who underwent F/B EVAR for complex thoracoabdominal aortic aneurysms between January 2018 and June 2024. The study investigated risk factors leading to reintervention, the association between reintervention and postoperative complications and mortality, and other related aspects.

**Results:**

A total of 103 patients were included, with 21 patients undergoing a total of 35 reinterventions during the follow-up period. Among these, 19 reintervention events occurred within 12 months after F/B EVAR (19/103, 18.4%). The reintervention group had significantly higher rates of hypertension, prior endovascular surgery, larger maximum aneurysm diameters, longer operative durations, and more postoperative ICU days compared to the non-reintervention group (*P* < 0.05). Hypertension (OR: 10.239, 95% CI: 0.999–104.916), maximum aneurysm diameter (OR: 1.591, 95% CI: 1.035–2.446), and operative duration (OR: 1.010, 95% CI: 1.004–1.017) were independent risk factors for reintervention. The most common reintervention methods were SMA branch stent implantation (4/35, 11.4%) and embolization of aortic endoleaks (4/35, 11.4%). The primary indication for reintervention was type IIIc endoleak (12/35, 34.3%). Most patients undergoing reintervention were discharged after interventional or open treatment, with 2 deaths post-intervention (2/21, 9.5%), a higher mortality rate than the non-reintervention group (3/82, 3.7%), but the difference was not statistically significant (*P* = 0.269).

**Conclusion:**

Hypertension, maximum aneurysm diameter, and operative duration were independent risk factors for reintervention. Type IIIc endoleak was the primary indication for unplanned postoperative reintervention, and interventional treatment was the most common reintervention method. Early postoperative follow-up is crucial, and personalized follow-up strategies and surgical approach selection are key to improving long-term prognosis.

## 1 Introduction

In recent years, fenestrated/branched endovascular aortic repair (F/B EVAR) has been widely applied to complex aortic lesions such as juxtarenal abdominal aortic aneurysm (JRAAA) and thoracoabdominal aortic aneurysm (TAAA). Compared with parallel stent techniques like chimney and periscope, F/B EVAR offers advantages such as physiological anatomic conformity and higher patency rates of branch vessels (1–3). However, physician-modified endografts (PMEG) have unique complications due to their stent-graft junction structures, such as endoleak between the main stent and the bridging stent of the branches (Type IIIc endoleak). Multiple studies have reported high reintervention rates after F/B EVAR, ranging from 10% to 40%, mostly related to its complications (4–12).

Current research predominantly focuses on outcomes of standardized EVAR devices, while long-term follow-up data for PMEGs remain scarce. International studies have elucidated correlations between endoleak classification and reintervention rates (13–17), yet systematic analyses of PMEG techniques are lacking. In domestic clinical practice, PMEG has gained widespread adoption due to its accessibility, but consensus remains elusive regarding postoperative reintervention patterns, risk factors, and optimization strategies. Furthermore, previous studies have prioritized technical success rates over in-depth exploration of independent predictors for reintervention, such as hypertension control and aneurysm diameter thresholds.

Our center began performing F/B EVAR surgeries in 2018, accumulating extensive surgical experience and collecting rich surgical and follow-up data. This study aims to systematically analyze clinical characteristics and risk factors of reintervention following PMEG-F/B EVAR in a single-center cohort. Specific objectives include elucidating the mechanistic impacts of risk factors on reintervention risks, ultimately proposing risk-stratified preoperative assessment, intraoperative optimization, and postoperative surveillance protocols to inform evidence-based clinical practice.

## 2 Materials and methods

### 2.1 Study design

#### 2.1.1 Case selection for inclusion

A retrospective review was conducted of all patients who underwent F/B EVAR surgery in the vascular surgery department of our center to screen consecutive cases of thoracoabdominal aortic aneurysm (TAAA) from January 2018 to June 2024.

(1) Inclusion Criteria:

Diagnosed with true aortic aneurysm with a diameter ≥5.0 cm or growth ≥0.5 cm/year involving important visceral branches;

Pre-rupture aneurysm involving visceral branches or inadequate anchoring zones;

Aortic aneurysm not involving the visceral artery region but with proximal or distal anchoring zones <1.5 cm from the visceral arteries.

(2) Exclusion Criteria:

Refusal to participate in follow-up or inability to comply with regular follow-up;

No preoperative computed tomography angiography (CTA) performed at our center;

Advanced malignant tumor with an expected life expectancy <2 years;

Participation in other clinical trials or use of any experimental drugs;

Concurrent congenital vascular malformations and connective tissue diseases.

All cases adhered to the above inclusion and exclusion criteria. This study was approved by the Ethics Committee of Nanjing Drum Tower Hospital (Approval No: 2022-678-02). Patients and their families were informed of the implementation plan and signed the surgical informed consent form preoperatively.

All patients enrolled in the study received PMEG, with no implantation of commercial constructed grafts.

#### 2.1.2 Case data collection

General data, including age, gender, preoperative comorbidities, and maximum aneurysm diameter, were collected from the hospital's electronic medical record system. Preoperative comorbidities included coronary heart disease, stroke, hypertension, hyperlipidemia, diabetes, chronic obstructive pulmonary disease, renal disease, cancer, and smoking history. Patients underwent CTA of the thoracoabdominal or abdominal aorta upon admission. DICOM files were exported from the hospital's imaging system to the professional measurement software EndoSize with centerline function for measurement to obtain the maximum aneurysm diameter.

#### 2.1.3 Surgical data collection

The surgical approach is differentiated based on whether branch stents are sutured to the stent graft: Fenestrated Endovascular Aortic Repair (F EVAR) refers to cases where none of the fenestrations are equipped with branch stents; Branched Endovascular Aortic Repair (B EVAR) refers to cases where fenestrations are equipped with branch stents.

Surgical-related data, including the number of fenestrations (1, 2, 3, 4) in each FB EVAR surgery, the number of branch vessels with implanted stents, the number of implanted branch stents, operation duration, postoperative ICU stay, and total postoperative hospital stay, were collected from the hospital's electronic medical record system.

#### 2.1.4 Follow-up methods and data collection

All patients were required to participate in follow-up visits at 1 month and 6 months post-discharge, and annually thereafter. The primary follow-up content was thoracoabdominal aortic CTA. The follow-up endpoint was patient death. Personalized follow-up protocols should be established according to individual patient conditions: For instance, patients with comorbid hypertension and/or diabetes should be referred to the endocrinology department during follow-up to maintain optimal blood pressure and glycemic control; patients developing postoperative vascular-related complications should undergo CTA re-evaluation following reintervention rather than adhering to annual CTA surveillance. Patient review records and reintervention records were collected from the hospital's electronic medical record system.

Reintervention Data Collection:

For patients undergoing one or multiple reinterventions, the following data were collected: reintervention time (perioperative: 0–30 days; short-term: 31–180 days; mid-term: >180 days), method (interventional/open), indication, length of hospital stay, and morbidity/mortality. Reintervention success was defined as the resolution of the indication.

### 2.2 Study endpoints and definitions

The study endpoints were defined as: Number of reinterventions, Reintervention, including perioperative and follow-up reinterventions, Death, Technical success.

Technique success was defined as successful access to the diseased segment of the aorta, placement of the aortic stent, accurate alignment of the fenestration, deployment of guidewire and catheter, implantation of branch stents with maintenance of blood flow in all target vessels, angiography demonstrating absence endoleaks, and patency maintained in all stent grafts.

Reintervention was defined as any unplanned surgery related to the aneurysm, device, or target artery. For patients undergoing more than one reintervention, each intervention was considered an independent event.

### 2.3 Statistical methods

Statistical analysis was performed using SPSS 26.0 software. Normality tests were conducted for measurement data. Measurement data that followed a normal distribution were expressed as mean ± standard deviation $\left(\bar{x}\pm s\right)$, while those that did not follow a normal distribution were expressed as median (M) with the first quartile (Q1) and third quartile (Q3). Count data were expressed as frequency (%). For measurement data that followed a normal distribution and had equal variances, parametric tests were used; otherwise, rank-sum tests were employed. Count data were analyzed using the *χ*^2^ test or Fisher's exact test. A *P*-value < 0.05 was considered statistically significant.

To identify independent risk factors for postoperative complications, multivariable logistic regression analysis was performed. Univariate analysis: Parametric tests were used for normally distributed measurement data with homogeneity of variance; otherwise, non-parametric rank sum tests were applied. χ^2^ tests or Fisher's exact tests were used for count data. Variables with *P* < 0.1 were recorded. Multivariate analysis: Variables screened from univariate analysis were included in logistic regression. Results reported regression coefficient (B), standard error (SE), Wald *χ*^2^ value, odds ratio (OR), significance level (P-value), and 95% confidence interval (95% CI).

## 3 Results

### 3.1 General information

A total of 103 patients who underwent F/B EVAR between January 2018 and June 2024 were included in this study. Among them, 21 patients received one or multiple reinterventions, while 82 patients did not receive any reintervention. Their baseline data are presented in Table 1. The incidence of hypertension was higher in the reintervention group compared to the non-reintervention group (*P* = 0.022 < 0.05). Patients in the reintervention group also had a higher proportion of prior endovascular surgical history (*P* = 0.034 < 0.05). Additionally, the aneurysm diameter was larger in the reintervention group than in the non-reintervention group, with a statistically significant difference (*P* = 0.023 < 0.05).

**Table 1:** General information of 103 patients treated with F/B EVAR.

Item	Overall (*n* = 103)	No intervention (*n* = 82)	Re-intervention (*n* = 21)	*p*-value
Male (*n*, %)	82 (79.6%)	67 (81.7%)	15 (71.4%)	0.209
Age [years, M (Q1, Q3)]	68 (60, 77)	68 (60, 77)	69 (57.5, 60)	0.977
Risk Factors (*n*, %)				
Hypertension	79 (76.7%)	59 (72.0%)	20 (95.2%)	0.022
Smoking	9 (8.7%)	8 (9.8%)	1 (4.8%)	0.470
Coronary Heart Disease	17 (16.5%)	14 (17.1%)	3 (14.3%)	0.759
Cerebral Infarction	8 (7.8%)	5 (6.1%)	3 (14.3%)	0.211
Renal Insufficiency	11 (10.7%)	9 (11%)	2 (9.5%)	0.848
Diabetes	15 (14.6%)	13 (15.9%)	2 (9.5%)	0.463
Hyperlipidemia	2 (1,9%)	2 (2.4%)	0	0.470
Chronic Obstructive Pulmonary Disease	2 (1.9%)	1 (1.2%)	1 (4.8%)	0.294
Malignant Tumor	2 (1.9%)	1 (1.2%)	1 (4.8%)	0.294
Aneurysm Maximum Diameter [cm, M (Q1, Q3)]	5.26 (4.54, 6.26)	5.07 (4.47, 5.88)	6.12 (4.79, 6.81)	0.023
Prior Surgical History (*n*, %)				
Open Surgery	6 (5.8%)	5 (6.1%)	1 (4.8%)	0.816
Endovascular Surgery	21 (20.4%)	13 (15.9%)	8 (3.8%)	0.034

Previous interventional surgery: Any endovascular procedure performed prior to the index F/B EVAR, excluding reinterventions for complications arising postoperatively.

M, represents the median; Q1, represents the first quartile; and Q3, represents the third quartile.

### 3.2 Surgical data

**Figure 1:**
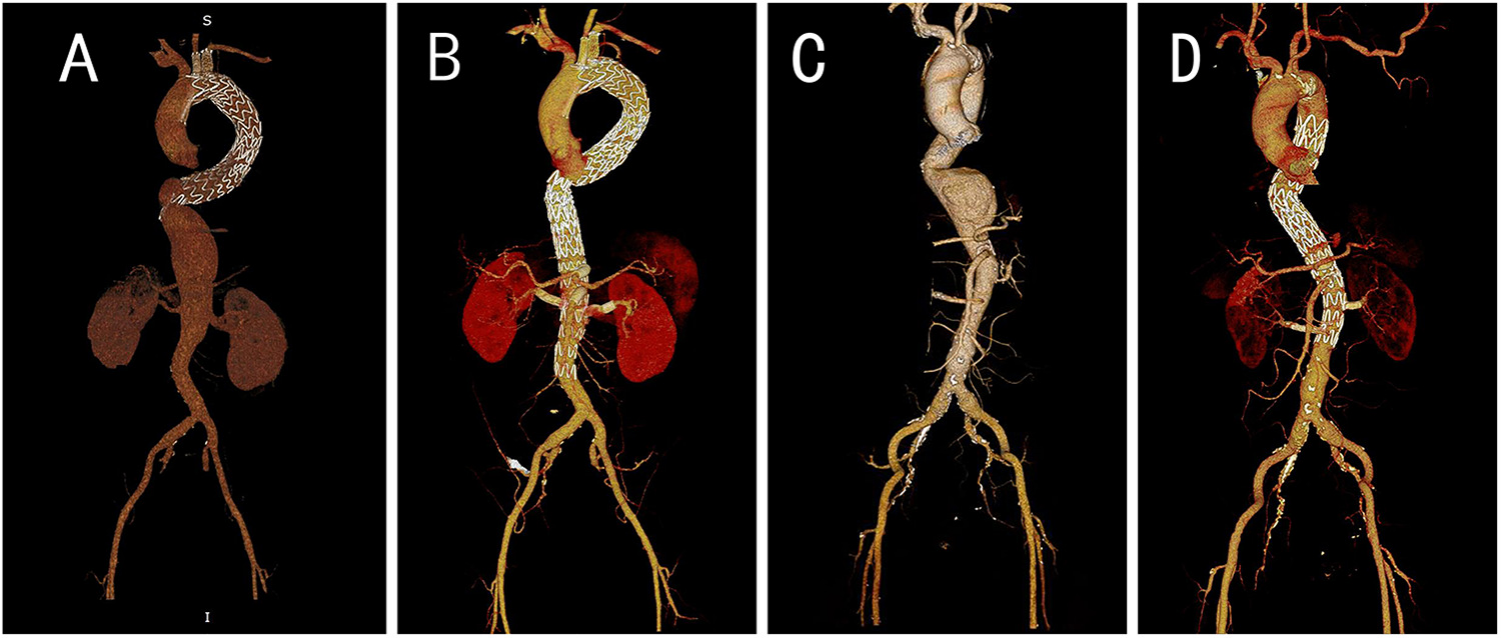
Pre- and post-operation CTA images for B EVAR and F EVAR. **(A)** Preoperative CTA of a patient with an abdominal aortic dissection aneurysm, revealing a giant false lumen, which led us to select B EVAR. **(B)** Postoperative CTA of the same patient. **(C)** Preoperative CTA of a patient with a thoracoabdominal aortic aneurysm, demonstrating an eccentric aneurysm, we opted for F EVAR. **(D)** Postoperative follow-up CTA of this patient.

Among the 103 patients in this study, 55 underwent F EVAR, and 48 underwent B EVAR. There was no significant difference in the surgical approaches employed between the reintervention group and the non-reintervention group. Figure 1 demonstrates the preoperative and postoperative CTA of F EVAR and B EVAR.

Compared to the non-reintervention group, the reintervention group had longer surgical durations, with a statistically significant difference (*P* = 0.000 < 0.05), and longer ICU stays (*P* = 0.005 < 0.05). The average number of fenestrations, the proportion of cases with three or four fenestrations, the average number of reconstructed branches, the average number of implanted branch stents, the total postoperative hospital stay, and the overall mortality rate were higher in the reintervention group than in the non-reintervention group, although these differences were not statistically significant (*P* > 0.05). Detailed data are shown in Table 2.

**Table 2:** Surgical data of 103 patients undergoing F/B EVAR treatment.

Item	Total (*n* = 103)	No reintervention (*n* = 82)	Reintervention (*n* = 21)	*p*-value
Surgical types (cases, %)				
F EVAR	55 (53.4%)	44 (53.7%)	11 (52.4%)	0.917
B EVAR	48 (46.6%)	38 (46.3%)	10 (47.6%)	0.917
Number of Fenestrations (cases, %)				
1	13 (12.6%)	10 (12.2%)	3 (14.3%)	0.797
2	16 (15.5%)	15 (18.3%)	1 (4.8%)	0.127
3	33 (32.0%)	25 (30.5%)	8 (38.1%)	0.505
4	41 (39.8%)	32 (39.0%)	9 (42.9%)	0.749
Average Number of Fenestrations (*x* ± s)	3.04 ± 1.02	2.99 ± 1.04	3.24 ± 0.94	0.317
Average Number of Reconstructed Branches (*x* ± s)	2.75 ± 0.97	2.70 ± 0.97	2.95 ± 0.97	0.279
Average Number of Implanted Branch Stents (*x* ± s)	3.05 ± 1.07	3.00 ± 1.09	3.24 ± 1.00	0.365
Operation Duration (min, *x* ± s)	314.43 ± 104.30	292.51 ± 92.27	400.00 ± 106.51	0.000
Postoperative ICU Stay Duration (days, *x* ± s)	1.17 ± 1.90	0.90 ± 0.73	2.19 ± 3.86	0.005
Total Postoperative Hospital Stay Duration (days, *x* ± s)	6.09 ± 2.44	6.07 ± 2.27	6.14 ± 3.09	0.908

### 3.3 Analysis of risk factors for reintervention

Univariate and multivariate analyses were conducted on the items that showed significant differences between the reintervention and non-reintervention groups in both the general and surgical data. Detailed results are presented in Table 3**.**

**Table 3:** Multivariate logistic regression analysis of independent risk factors for reintervention in patients undergoing F/B EVAR during follow-up.

Factor	B	SE	Wald *χ*^2^	*P*-value	OR	95% CI
Hypertension	2.326	1.187	3.839	0.050	10.239	0.999–104.916
Maximum Aneurysm Diameter (cm)	0.465	0.219	4.481	0.034	1.591	1.035–2.446
Prior Endovascular Surgery	1.158	0.668	3.005	0.083	3.183	0.860–11.785
Operation Duration (min)	0.010	0.003	9.853	0.002	1.010	1.004–1.017
Postoperative ICU Stay Duration (days)	0.279	0.227	1.518	0.218	1.322	0.848–2.061

The results indicated that a history of hypertension, the maximum preoperative aneurysm diameter, and surgical duration were independent risk factors for postoperative reintervention (*P* ≤ 0.05). Specifically, the odds ratio (OR) for hypertension was 10.239 (0.999–104.916), with *P* = 0.05, meaning that patients with a history of hypertension had a 10.239-fold higher risk of postoperative reintervention compared to those without. The OR for the maximum aneurysm diameter was 1.591 (1.035–2.446), with *P* = 0.034, indicating that for every 1 cm increase in the preoperative aneurysm diameter, the risk of postoperative reintervention increased by 59.1%. The OR for surgical duration was 1.010 (1.004–1.017), with *P* = 0.002, suggesting that for every 1-minute increase in surgical duration, the risk of postoperative reintervention increased by 1%.

### 3.4 Reintervention outcomes

The average follow-up time was 41.77 ± 23.30 months, with 40.17 ± 23.97 months for the non-reintervention group and 48.00 ± 19.78 months for the reintervention group. In the reintervention group, 21 patients underwent a total of 35 reinterventions. The types of reinterventions are listed in Table 4, and the indications for reintervention are presented in Table 5.

**Table 4:** Classification and incidence of 35 reinterventions in 21 patients.

Classification of reinterventions	Total	Postoperative <30 days	30–180 days	>180 days
Branch Stent Implantation (*n* = 8)				
SMA	4	1	1	2
LRA	3	1	1	1
RRA	1	1		
Endoleak Embolization (*n* = 11)				
LRA	3	1	2	
RRA	2			2
Lumbar Artery	2		1	1
Aorta	4		1	3
Aorto-iliac Stent Implantation (*n* = 11)				
Internal Iliac Artery Embolization + Iliac Stent	2	1		1
Thoracic Aortic Stent	3	2	1	
Abdominal Aortic Stent	2	1		1
Abdominal Aortic + Iliac Stent	1	1		
Aortic Cuff Stent	2			2
Abdominal Aortic Stent + Endoleak Embolization	1			1
Open Surgery (*n* = 5)				
Abdominal Aortic Aneurysmectomy + Endoleak Repair	1			1
Thoracoabdominal Aortic Aneurysmectomy	1			1
Femoral Artery Pseudoaneurysmectomy	2	2		
Lower Extremity Arterial Thrombectomy	1	1		
Total	35	12 (34.3%)	7 (20.0%)	16 (45.7%)

**Table 5:** Indications for 35 reinterventions in 21 patients.

Reintervention indications	Total	Postoperative <30 days	30–180 days	>180 days
Endoleak				
Type Ia	2		2	
Type Ib	8	1		7
Type Ic	3	2		1
Type II	2		1	1
Type IIIa	2			2
Type IIIc	12	4	4	4
Branch Occlusion	1			1
TBAD	2	2		
Pseudoaneurysm	2	2		
Lower Extremity Arterial Thrombosis	1	1		
Total	35	12 (34.3%)	7 (20.0%)	16 (45.7%)

The most common types of reinterventions were SMA branch stent implantation (4/35, 11.4%) and aortic endoleak embolization (4/35, 11.4%), followed by LRA stent implantation (3/35, 8.6%) and LRA endoleak embolization (3/35, 8.6%). The number of branch artery-related reinterventions (stent implantation + embolization) totaled 13 times (37.1%), which was less than the number of main-iliac artery-related reinterventions (stent implantation + embolization + open surgery) at 17 times (48.6%). Most reinterventions occurred in the perioperative period (12/35, 34.3%) and the mid-term (16/35, 45.7%).

Among the 35 reinterventions included in this study, the primary indication for reintervention was endoleak (29/35, 82.9%), with the most common being type IIIc endoleak (12/35, 34.3%) and type Ib endoleak (8/35, 22.9%). The incidence of type IIIc endoleak was equal across all periods, while type Ib endoleak mainly occurred in the mid-term. Overall, the incidence of type I and type III endoleaks was similar (13 vs. 14), and type II endoleak occurred twice (2/35, 5.7%). Both TBAD (2/35, 5.7%) and pseudoaneurysm (2/35, 5.7%) occurred in the perioperative period. The incidence of branch occlusion (1/35, 2.9%) and lower extremity arterial thrombosis (1/35, 2.9%) was low.

Among the 21 patients who underwent reintervention, 2 patients underwent 4 reinterventions (4/21, 9.5%), 3 patients underwent 3 reinterventions (3/21, 14.3%), 2 patients underwent 2 reinterventions (9.5%), and 14 patients underwent 1 reintervention (16/21, 66.7%). The average number of reinterventions was 1.67. The average postoperative hospital stay after reintervention was 5.23 days, with the longest stay being 61 days. Two patients died after surgery, with detailed information provided in Table 6.

**Table 6:** Surgical data and prognosis of 35 reinterventions in 21 patients.

Patient No.	Reintervention type	Indication	Open/interventional	Postoperative hospital stay	Death
1	LRA Endoleak Embolization	Type IIIc Endoleak	Interventional	1	–
	RRA Endoleak Embolization	Type IIIc Endoleak	Interventional	2	–
	LRA Stent Implantation	Type IIIc Endoleak	Interventional	1	–
2	Left Lower Extremity Arterial Thrombectomy	Acute Lower Extremity Arterial Thrombosis	Open	4	–
3	SMA Stent Implantation	Type IIIc Endoleak	Interventional	3	–
4	LRA Endoleak Embolization	Type IIIc Endoleak	Interventional	1	–
5	Aortic Endoleak Embolization	Type Ia Endoleak	Interventional	5	–
	SMA Stent Implantation	Type IIIc Endoleak	Interventional	3	–
	LRA Stent Implantation	Type IIIc Endoleak	Interventional	3	–
	Aortic Stent Implantation	Type Ia Endoleak	Interventional	4	–
6	Abdominal Aortic Stent + Endoleak Embolization	Type Ib Endoleak	Interventional	3	–
7	Thoracoabdominal Aortic Aneurysm Neck Ligation	Type IIIa Endoleak	Open	8	–
8	Thoracic Aortic Stent	TBAD	Interventional	3	–
9	SMA Stent Implantation	SMA Occlusion	Interventional	2	–
	Lumbar Artery Embolization	Type II Endoleak	Interventional	1	–
10	Internal Iliac Artery Embolization + Iliac Stent	Type Ic Endoleak	Interventional	4	–
11	Aortic Cuff Stent	Type IIIa Endoleak	Interventional	3	–
12	Thoracic Aortic Stent	TBAD	Interventional	5	–
13	LRA Stent Implantation	Type IIIc Endoleak	Interventional	1	–
	RRA Stent Implantation	Type IIIc Endoleak	Interventional	3	–
	Abdominal Aortic + Iliac Stent	Type Ic Endoleak	Interventional	2	–
14	Pseudoaneurysmectomy	Femoral Artery Pseudoaneurysm	Open	25	–
	Abdominal Aortic Aneurysmectomy + Endoleak Repair	Type Ib Endoleak	Open	61	–
	Aortic Cuff Stent	Type IIIa Endoleak	Interventional	2	Death on Day 2 Postop
15	LRA Stent Implantation	Type IIIc Endoleak	Interventional	3	–
16	RRA Endoleak Embolization	Type IIIc Endoleak	Interventional	1	–
	Aortic Endoleak Embolization	Type Ib Endoleak	Interventional	2	–
	SMA Stent Implantation	Type IIIc Endoleak	Interventional	1	–
	Aortic Endoleak Embolization	Type Ib Endoleak	Interventional	2	–
17	Aortic Endoleak Embolization	Type Ib Endoleak	Interventional	1	Death on Day 1 Postop
18	Abdominal Aortic Stent	Type Ib Endoleak	Interventional	6	–
19	Lumbar Artery Embolization	Type II Endoleak	Interventional	2	–
20	Abdominal Aortic Stent	Type Ib Endoleak	Interventional	5	–
	Internal Iliac Artery Embolization + Iliac Stent	Type Ic Endoleak	Interventional	3	–
21	Pseudoaneurysmectomy	Femoral Artery Pseudoaneurysm	Open	7	–

In this study, aortic stent graft we used included Zenith (Cook Medical, US), Ankura (Lifetech Scientific, China), Valiant Captivia (Medtronic, US), and Hercules (MicroPort, China). Branch stent included Viabahn (Gore, US), Fluency (Bard, US), Omnilink Elite (Abbott, US), Everflex (Medtronic, US) and SMART (Cordis, US). The most frequently used aortic stent graft was Ankura, while the predominantly employed branch stent graft was Viabahn. Brand of grafts were provided in Table 7.

**Table 7:** Brand of grafts.

Item	Total	No Reintervention	Reintervention
Aortic Stent Graft (*n*, %)	113 (100%)	88 (100%)	25 (100%)
Zenith (Cook Medical, US)	24 (21.2%)	18 (20.5%)	6 (24.0%)
Ankura (Lifetech scientific, China)	66 (58.4%)	53 (60.2%)	13 (52.0%)
Valiant Captivia (Medtronic, US)	3 (2.7%)	1 (1.1%)	2 (8.0%)
Hercules (MicroPort, China)	20 (17.7%)	16 (18.2%)	4 (16.0%)
Branch Stent (*n*, %)	314 (100%)	246 (100%)	68 (100%)
Viabahn (Gore, US)	171 (54.5%)	140 (57.0%)	31 (45.6%)
Fluency (Bard, US)	84 (26.5%)	55 (22.4%)	29 (42.6%)
Omnilink Elite (Abbott, US)	51 (16.2%)	43 (17.5%)	8 (11.8%)
Everflex (Medtronic, US)	3 (1.0%)	3 (1.2%)	0 (0%)
SMART (Cordis, US)	5 (1.6%)	5 (2.0%)	0 (0%)

### 3.5 Other follow-up results

A total of 82 patients did not receive reintervention treatment. The average follow-up time for the non-reintervention group was 40.17 ± 23.97 months. Postoperative complications included spinal ischemia (3/82, 3.7%), renal function decline (5/82, 6.1%), cerebral complications (3/82, 3.7%), and respiratory/digestive system complications (2/82, 2.4%). There was 1 perioperative death (1/82, 1.2%) and 2 deaths during follow-up (2/82, 2.4%), with no aortic-related deaths. The overall mortality rate in the non-reintervention group (3/82, 3.7%) was lower than that in the reintervention group (2/21, 9.5%), but the difference was not significant (*P* = 0.269).

## 4 Discussion

F/B EVAR has expanded the indications for fully endovascular treatment of complex aortic lesions. It has evolved from an alternative treatment for elderly or high-risk patients to a first-line treatment suitable for the majority of anatomical structures, and has even become one of the primary choices in emergency situations (1–3). PMEG can adapt to complex individual anatomical structures and maintain blood flow to vital organs (18), but this personalized design may increase the risk of postoperative complications and unplanned reinterventions (19).

Statistical results from the study indicate that a history of hypertension, the maximum diameter of the aortic aneurysm, and surgical duration are independent risk factors for reintervention. In line with the experience of the surgeons in this group, patients with poor postoperative blood pressure control and larger aortic aneurysm diameters often have a higher risk of endoleak and stent migration. Surgical duration is usually correlated with the number of reconstructed branches, and as the number of reconstructed branches increases, the risk of endoleak at the junction between the branches and the main stent also increases, which may explain the increased risk of reintervention due to longer surgical duration. A history of prior endovascular surgery also shows marginal significance. During surgery, patients with existing proximal stents tend to avoid upper limb access. For patients requiring multiple fenestrations, the most commonly used method in our center is to place a large sheath (>16F) in the femoral artery, and then puncture a conventional vascular sheath on the large sheath to protect the vascular endothelium and control bleeding at the access site.

In this study the re-intervention rate after F/B EVAR was 20.4%, almost half of the reinterventions occurred within 180 days, which is similar to the result obtained by Sénémaud J (20). The most common indications for reintervention in this study were type Ⅲc endoleak and type Ⅰb endoleak. Reinterventions due to various types of endoleak accounted for 82.9% of the total, indicating that endoleak is the primary indication for postoperative intervention after F/B EVAR. Branch artery occlusion was relatively rare, with only one case, suggesting a high patency rate of branches in F/B EVAR.

For indications primarily involving endoleak, interventional surgery is usually the preferred choice when selecting a reintervention plan. which is consistent with the findings of Tachida A et al. (21). Compared to traditional open surgery, interventional surgery has the advantages of being minimally invasive and having a faster recovery, allowing patients to resume daily activities in a shorter period of time. Based on this, enabling most patients to undergo reintervention in an outpatient surgical setting may effectively reduce patient burden. By optimizing the treatment process and improving the accessibility of outpatient surgery, more efficient and economical medical services can be provided to patients, thereby improving overall treatment outcomes and patient satisfaction.

While the minimally invasive advantages of interventional surgery are impressive, its therapeutic certainty is lacking. Among the 21 patients who underwent reintervention, 7 (33.3%) underwent two or more interventional surgeries, and 2 underwent four interventional surgeries, highlighting the necessity for regular follow-up after reintervention. However, repeated interventional procedures such as endoleak embolization pose significant economic and psychological challenges for patients. Improving the reliability of existing interventional techniques and exploring new minimally invasive reintervention methods may be reasonable ways to improve the prognosis of patients undergoing reintervention. Open surgery is mostly used for vascular access-related complications, such as pseudoaneurysms, lower extremity thrombosis, and complications that cannot be resolved through interventional methods. Patient No. 7 had a reintervention indication of type Ⅲa endoleak, where the two sections of the aortic stent formed an angle at the junction between the SMA and LRA, making it impossible to completely embolize with coils and too narrow to implant a cuff stent. Therefore, an open abdominal surgery was performed to ligate the aneurysm neck to eliminate the aneurysmal sac.

Despite the possibility of reintervention, the advantages of F/B EVAR in the treatment of complex aortic aneurysms still make it a first-line treatment option (22). Patients in this study underwent high-precision imaging measurements before surgery (aortic CTA with an accuracy of within 1 mm), using professional measurement software such as EndoSize (Therenva SAS, Rennes, France), which have functions such as marking the centerline, 3D reconstruction, and multiplanar reconstruction, to measure the anatomical and morphological characteristics of the aorta and its branch arteries.

Currently, F/B EVAR can only be performed in a few experienced centers, mainly because the customization of stents and intraoperative branch positioning require extremely high precision and significant experience for surgeons. Despite comprehensive preoperative planning, less experienced surgeons may still encounter more technical difficulties during the surgery. Since 2018, our center has been implementing a structured training program, using 3D-printed aneurysm models for simulation training and encouraging experienced surgeons to guide new surgeons in their operations. These measures may have mitigated this impact.

As a complex endovascular repair technique, F/B EVAR has been subject to concern due to its relatively high postoperative reintervention rate. Our objective is to systematically collect comprehensive clinical data to facilitate early identification of potential reintervention risks, optimize both surgical approaches and perioperative management protocols, and tailor individualized follow-up strategies. Through these initiatives, we aim to achieve a sustained reduction in post-F/B EVAR reintervention rates, with the overarching goal of improving long-term patient prognosis. For instance, in hypertensive patients, intensive perioperative blood pressure management should be implemented with systolic blood pressure controlled around 130 mmHg. For patients with branch artery opened on the aneurysm sac, side branch may be utilized to enhance sealing and reduce type Ⅲc endoleak risks. High-risk patients with reintervention risk factors should undergo intensified first-year surveillance: CTA follow-up examinations were conducted at the first postoperative month, then at 3 months and 6 months postoperatively, followed by annual CTA evaluations thereafter. Routine patients follow standard follow-up schedules.

This research has certain limitations. The retrospective design inherently carries risks of selection bias, potentially constraining the generalizability of our findings—a limitation that future multicenter prospective trials could address to strengthen the evidence hierarchy. Furthermore, the extended time span of this study introduces potential confounders, such as evolving surgical proficiency, which may have influenced patient outcomes. Besides, long-term follow-up studies are warranted to address these factors.

## 5 Conclusion

This study conducted a retrospective review of a single-center experience with F/B EVAR for treating complex thoracoabdominal aortic aneurysms, revealing that Type IIIc endoleak was the primary cause of the high reintervention rate following F/B EVAR. Hypertension, maximum aneurysm diameter, and prolonged operative time were identified as independent risk factors for reintervention. A personalized management and follow-up may be key to the timely management of complications and improved patient outcomes.

## Data Availability

The original contributions presented in the study are included in the article, further inquiries can be directed to the corresponding author.
